# Supplementary material for: Investigation on factors affecting early strength of high-performance concrete by Gaussian Process Regression
Source: PLoS One. 2022 Jan 27;17(1):e0262930. doi: 10.1371/journal.pone.0262930 (PMC8794196; doi:10.1371/journal.pone.0262930)
Supplement: S1 Appendix — (DOCX) [file pone.0262930.s001.docx]

S1 Appendix


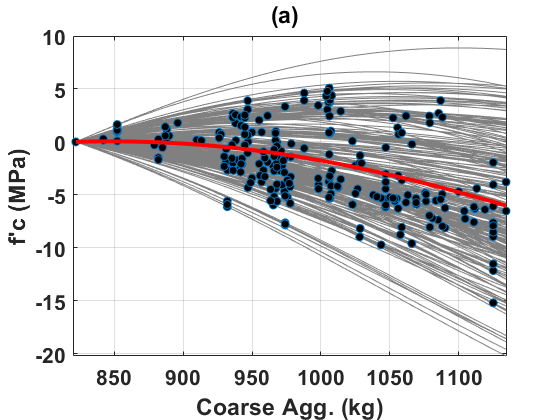

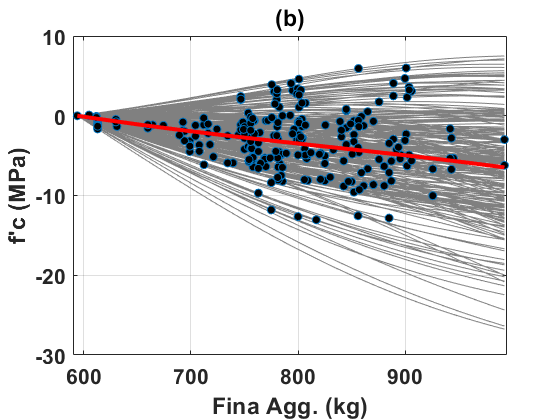


**Fig. A.** ICE and PDP curves in function of input variables with offset for (a) coarse aggregates; and (b) fine aggregates.
